# Supplementary material for: Identification of photoperiod-induced specific miRNAs in the adrenal glands of Sunite sheep (Ovis aries)
Source: Front Vet Sci. 2022 Jul 22;9:888207. doi: 10.3389/fvets.2022.888207 (PMC9354845; doi:10.3389/fvets.2022.888207)
Supplement: Supplementary file 1 [file Data_Sheet_1.ZIP › Supplementary file/miRDeep2 file.zip!/miRDeep2 file/Know_Structure/SP42A3/oar-mir-758.pdf]

miRBase precursor : oar-mir-758
Total read count : 244
oar-miR-758-5p read count : 0
oar-miR-758-3p read count : 242
remaining reads : 2

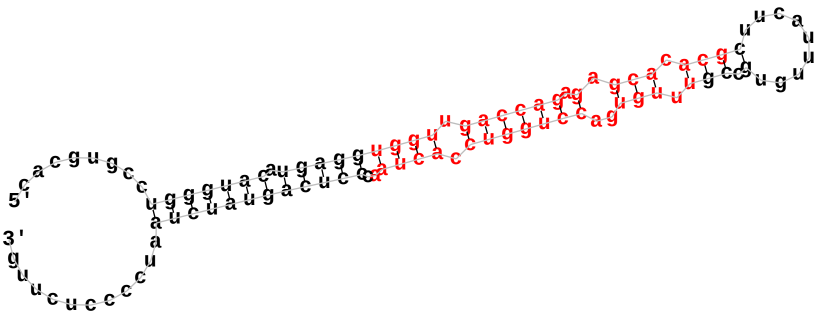

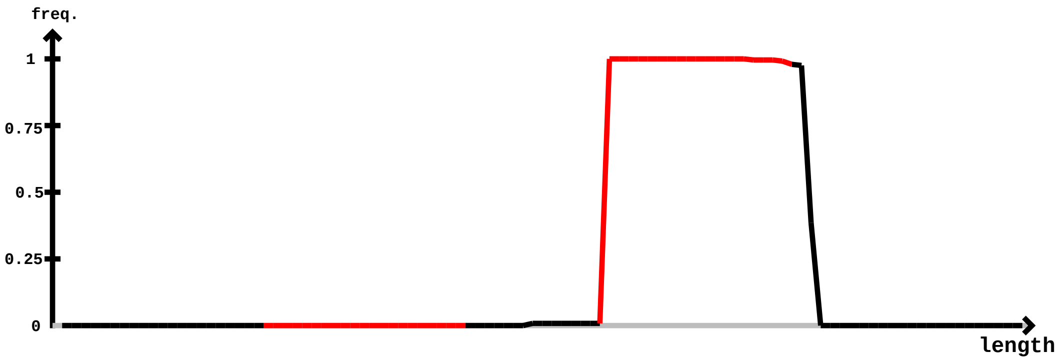


```
                                                    oar-miR-758-3p
                    oar-miR-758-5p
5'- cacgugccugggguacaugaggugguugaccagagagcacacgcuucauuugugccguuugugaccugguccacuaacccucaguaucuaauccccucuug   -3'    exp
    ........(((((((.(((((((((.((((((.(.(((.((((.........).))).)))..))))))).))))..))))))))))))...........    reads     mm      sample
    ................................................uugugccguuugugaccuggucc.............................    1         0       seq
    ................................................uugugccguuugugaccugguccacu..........................    1         0       seq
    ........................................................uuugugaccugguccacua.........................    3         0       seq
    ........................................................uuugugaccugguccacuaa........................    1         0       seq
    ........................................................uuugugaccugguccacuaac.......................    144       0       seq
    ........................................................uuugugaccugguccacuaacc......................    94        0       seq
```
